# A Biologically Plausible Architecture of the Striatum to Solve Context-Dependent Reinforcement Learning Tasks

**DOI:** 10.3389/fncir.2017.00045

**Published:** 2017-06-21

**Authors:** Sabyasachi Shivkumar, Vignesh Muralidharan, V. Srinivasa Chakravarthy

**Affiliations:** Computational Neuroscience Lab, Department of Biotechnology, Bhupat and Jyoti Mehta School of Biosciences, Indian Institute of Technology MadrasChennai, India

**Keywords:** striatum, basal ganglia, context dependent learning, striosomes and matrisomes, self organizing maps, modular reinforcement learning

## Abstract

Basal ganglia circuit is an important subcortical system of the brain thought to be responsible for reward-based learning. Striatum, the largest nucleus of the basal ganglia, serves as an input port that maps cortical information. Microanatomical studies show that the striatum is a mosaic of specialized input-output structures called striosomes and regions of the surrounding matrix called the matrisomes. We have developed a computational model of the striatum using layered self-organizing maps to capture the center-surround structure seen experimentally and explain its functional significance. We believe that these structural components could build representations of state and action spaces in different environments. The striatum model is then integrated with other components of basal ganglia, making it capable of solving reinforcement learning tasks. We have proposed a biologically plausible mechanism of action-based learning where the striosome biases the matrisome activity toward a preferred action. Several studies indicate that the striatum is critical in solving context dependent problems. We build on this hypothesis and the proposed model exploits the modularity of the striatum to efficiently solve such tasks.

## Introduction

In order to understand the role of the striatum within the basal ganglia (BG) circuit, it is essential to understand the rich and complex microcircuitry of this structure. It is well-known that the striatum has a modular architecture, containing specialized input-output structures called the “striosomes” and regions of the surrounding matrix called the “matrisomes” (Graybiel et al., 1991). The striosomes are known to receive limbic inputs and send their projections to the substantia nigra pars compacta, a midbrain dopaminergic nucleus, whereas the matrisomes mostly receive sensorimotor and associative inputs and project to downstream BG nuclei (Graybiel et al., 1994). The cortico-striatal connectivity seems to show a divergence property, where there is spread of connections coming from the cortex to the striatum followed by a convergence at the level of the globus pallidus (GP; Graybiel et al., 1994). There have also been suggestions that the striatum constructs low dimensional representations of the cortical states via the cortico-striatal projections (Bar-Gad et al., 2000, 2003). Indirect evidence for this comes from experiments which indicate hebbian like learning in cortico-striatal projections (Charpier and Deniau, 1997). Therefore, the striatum has the cellular and molecular machinery to possibly construct such reduced representations of cortical states. These facts about striatal microanatomy lead us to believe that the striatum could build representations for several state and action spaces.

Anatomically the striosome-matrisome complex has a center-surround structure (Graybiel et al., 1991), and the proposed computational architecture for the striatum is inspired by this fact. Studies investigating the projection of prefrontal areas to the striosomes show specificity to certain cortical areas (Eblen and Graybiel, 1995). These cortical projections to anterior striosomes are mostly from frontal regions like the orbitofrontal cortex, anterior insula, and the anterior cingulate cortex (Eblen and Graybiel, 1995) which could very well represent the task or state space (Wilson et al., 2014). The matrisome which receives more sensorimotor information would well represent the action space (Flaherty and Graybiel, 1994). In classical reinforcement learning (RL) literature, the expected reward signal in a given state is called the value function (Sutton and Barto, 1998). The striosomes are known to have reciprocal projections to both the ventral tegmental area (VTA) and the substantia nigra pars compacta (SNc) and thus would receive the prediction error signal from these midbrain nuclei, which can serve as a reinforcement signal that aids in the computation of the state value function (Granger, 2006; Wall et al., 2013). On the other hand the action representations perhaps evolve at the level of matrisomes, and get mapped on to action primitives at the level of GPi (Pasquereau et al., 2007). Thus, using the reward information from the environment and the representations built in the striatum, the BG can learn to perform reward based decision making tasks.

This functional organization and the modularity of the striatum has been hypothesized to perform context dependent tasks (Amemori et al., 2011). Mulitple spatio-temporal contexts could then be mapped to different striatal modules. This leads to the distribution of context information to different modules, a facet of modular reinforcement learning (Kalmár et al., 1999). We then consider the selection of the module appropriate to a given context to be driven by a responsibility signal, which is a function of the uncertainty in the environment. Uncertainty in the environment from previous approaches has been represented by reward variance (Balasubramani et al., 2015). Since change in context leads to increased uncertainty, reward variance could help identify this change.

In the current study, we propose a hierarchical self-organizing structure to model the striosome-matrisome compartments. Self-organizing maps (SOMs) have been used to represent high-dimensional information in 2-D sheets of neurons (Kohonen, 1990). The striosome and the matrisome layers are both modeled as a double SOM layer, consisting of Strio-SOM and Matri-SOM respectively, where a single Strio-SOM neuron has projections to the surrounding Matri-SOM neurons. The activity of the Matri-SOM is mapped to action primitives via the direct and indirect pathways of the BG to perform action selection. The reward information from the environment is utilized by the Strio-SOM to bias the surrounding Matri-SOM activity toward a preferred action. This provides a biologically plausible way of carrying out action based Q-learning (Sutton and Barto, 1998) and is a novel feature of our model. This model has been tested on standard grid-world problems.

The model has been extended to cater to problems with varying contexts (changing reward locations). Different striatal modules map different contexts and tonically active neurons (TANs; Apicella, 2007) aid in module selection This selection is driven by the risk (reward variance) in the environment which is used to calculate the responsibility signal (Amemori et al., 2011) for a particular module. We have tested this model on grid-world problems with varying reward distributions and the model is able to solve these problems efficiently.

## Methods

### Modeling the microanatomy of the striatum

**Figure 1:**
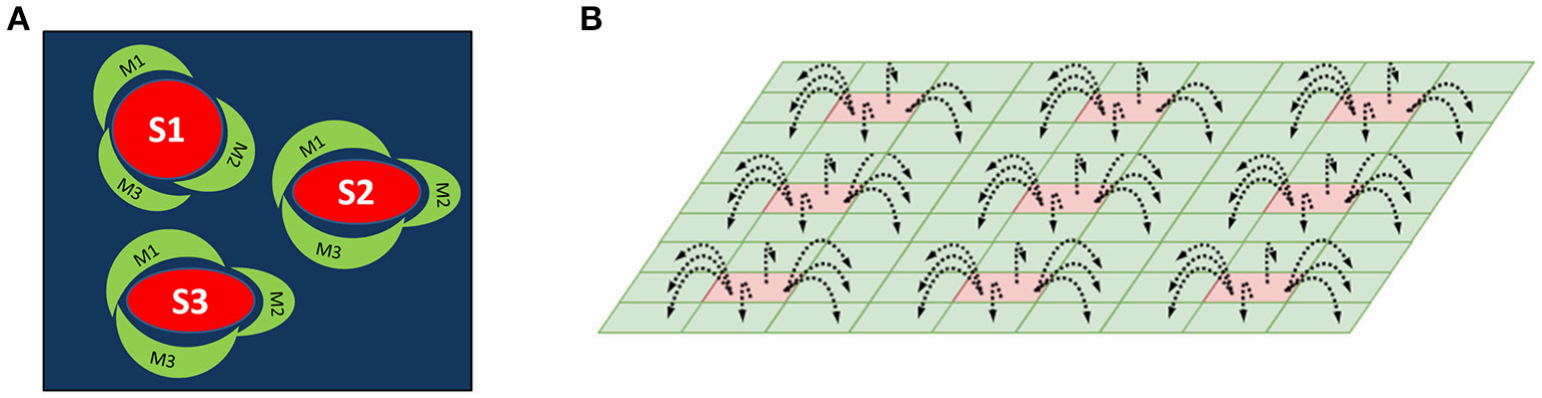
**(A)** A schematic of the striosome-matrisome center surround mapping in the striatum. The red structures represent the striosomes and the surrounding green structures represent the matrisomes. **(B)** A Schematic of the layered SOM structure modeling the striosomes and matrisomes. The Strio-SOM (Red) represents the striosomes and the Matri-SOM (Green) represents the matrisomes; each Strio-SOM neuron has projections to the surrounding Matri-SOM neurons.

We have proposed an architecture consisting of two layers of SOMs as a method for mapping center-surround structures seen in the striatum (Figure 1A). This architecture is used to model striosomes and matrisomes which map the state space and action space, respectively.

The first layer called Strio-SOM models the striosomes and maps the state space. The second layer activated by the Strio-SOM is called the Matri-SOM which models the matrisomes and maps the action space (Figure 1B).

In order to map the state space, we have a Strio-SOM of size *m*_1_ × *n*_1_. If *s* is a state vector, the weights of the Strio-SOM (*W*^*S*^) are of dimension *m*_1_ × *n*_1_ × dim(*s*), where dim(*s*) stands for the dimension of the state vector *s*. Similarly, to map the action space, we have a Matri-SOM of size *m*_2_ × *n*_2_. If *a* is an action vector, the weights of all the Matri-SOMs (*W*^*M*^) are of dimension *m*_1_ × *n*_1_ × *m*_2_ × *n*_2_ × dim(*a*) as each neuron in the Strio-SOM is connected to a Matri-SOM.

The activity for a neuron n in the Strio-SOM for a state input *s* is given in Equation (1).

(1) $${X^{S}}_{\left[n\right]}=\exp(\frac{-||{W^{S}}_{\left[n\right]}-s|{{|}_{2}}^{2}}{{\sigma_{S}}^{2}})$$

where [*n*] represents the spatial location of the neuron *n* and σ_S_ controls the sharpness of the neuron activity. The complete activity of the Strio-SOM (*X*^*S*^) is the combination of individual activity of all the neurons. The neuron with the highest activity (“winner”) for a state *s* is denoted by *n*_s_^*^**.

Similarly, the activity for a neuron n in the Matri-SOM for an action input *a* in a state *s* is given in Equation (2).

(2) $${X^{M}}_{\left[{n_{s}}^{*}][n\right]}=\exp(\frac{-||{W^{M}}_{\left[{n_{s}}^{*}][n\right]}-a|{{|}_{2}}^{2}}{{\sigma_{M}}^{2}})$$

where σ_M_ controls the sharpness of the neuron activity. The complete activity of the Matri-SOM corresponding to neuron *n*_s_^*^ (${X^{M}}_{\left[{n_{s}}^{*}\right]}$) is the combination of individual activity of all the neurons in the Matri-SOM corresponding to $n_{s}^{*}$. The neuron with the highest activity (“winner”) for an action *a* in a state *s* is denoted as *n*_s,a_^*^.

The weight of a neuron *n* in the Strio-SOM for a state input *s* is updated according to the following rule (Equation 3)

(3) $$W_{\left[n\right]}^{S}\leftarrow W_{\left[n\right]}^{S}+\eta_{S}.\exp(\frac{-||[n]-[{n_{s}}^{*}]|{{|}_{2}}^{2}}{{\sigma_{S}}^{2}}).(s-W_{\left[n\right]}^{S})$$

The weight of neuron *n* in the Matri-SOM for an action input *a* in a state *s* is updated according to Equation (4).

(4) $$\begin{array}{l} W_{\left[{n_{s}}^{*}][n\right]}^{M}\leftarrow W_{\left[{n_{s}}^{*}][n\right]}^{M}\\ +\eta_{M}.\exp(\frac{-||[n]-[{n_{s,a}}^{*}]|{{|}_{2}}^{2}}{{\sigma_{M}}^{2}}).(a-W_{\left[{n_{s}}^{*}][n\right]}^{M}) \end{array}$$

### Reinforcement learning in basal ganglia

**Figure 2:**
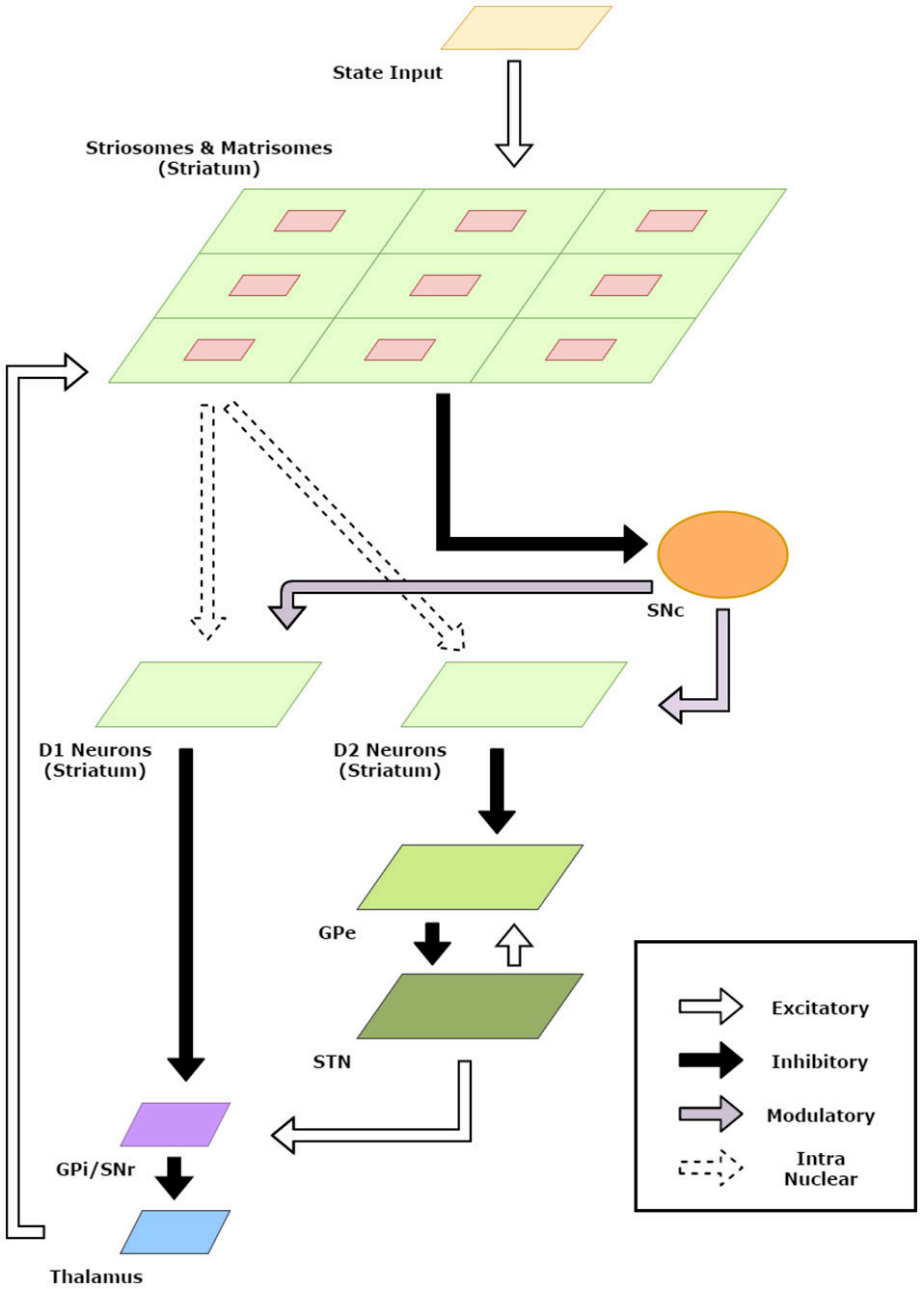
Schematic Diagram for the basal ganglia model. The arrows indicate connections and their type. The component sizes are proportional to their dimensions. The feedback connections from the thalamus project the information about the action chosen back to the striatum.

The striatum model developed in the previous section was useful in developing representations for states and actions. In this section, we incorporate the striatum model in a BG model and apply the model to standard reinforcement learning tasks. A schematic diagram of the model is given in Figure 2.

Let us assume that the animal is in a state *s*. The activity of the striosomes gives us the representation of the state in the striatum. In our model, the activity of the striosomes is given as the activity of the neurons in the Strio-SOM where the activity of a single neuron is given by Equation (1). Thus, the activity is of dimension *m*_1_ × *n*_1_.

This activity of the Strio-SOM projects to the SNc and represents the value for the state *s* in our model (Equation 5). These weights from the striatum to SNc (*W*^*Str* → *SNc*^) are trained using the signal from SNc which is representative of Temporal Difference (TD) error (δ) (Equation 6). The TD error is calculated as δ = *r* + γ*V*(*s*′) − *V*(*s*) where *s*′ is the new state after taking action *a* (Equation 19), *r* is the reward obtained and γ is the discount factor.

(5) $$V(s)=\sum_{\forall n}{W^{Str\to SNc}}_{\left[n\right]}{X^{S}}_{\left[n\right]}$$

(6) $$\Delta {W^{Str\to SNc}}_{\left[n\right]}=\eta^{Str\to SNc}\delta {X^{S}}_{\left[n\right]}$$

where *V*(*s*) represents the value for state *s*, η^*Str*→*SNc*^ is the learning rate for W^*Str*^^ → *SNc*^.

The representation for the various actions the agent in state *s* can perform is given by the activity of the matrisomes surrounding the corresponding striosome neuron for the state. In our model, this is given by the activity of the neurons of the Matri-SOM corresponding to the neuron with the highest activity in the Strio-SOM (*n*_s_^*^) where the activity of a single neuron in the Matri-SOM is given in Equation (2). Thus, the activity is of dimension *m*_2_ × *n*_2_. The action input *a* is given as feedback input from the thalamus to the striatum (Figure 2).

The activity of Matri-SOM neurons is further tuned by the connections between the neurons in the Strio-SOM and the Matri-SOM (*W*^*S*→*M*^). These connections are also trained using TD error as above using the Matri-SOM activity for the action (*a*) chosen, as follows:

(7) $$\begin{array}{l} {Y^{M}}_{\left[{n_{s}}^{*}][n\right]}=\alpha {X^{M}}_{\left[{n_{s}}^{*}][n\right]}\\ \;+(1-\alpha ){W^{S\to M}}_{\left[{n_{s}}^{*}][n\right]}{X^{S}}_{\left[{n_{s}}^{*}\right]} \end{array}$$

(8) $$\Delta {W^{S\to M}}_{\left[{n_{S}}^{*}][n\right]}=\eta^{S\to M}\delta {X^{M}}_{\left[{n_{S}}^{*}][n\right]}$$

where α controls the contribution of the action and the lateral connections to the activity of the Matri-SOM and η^*Str*→*SNc*^ is the learning rate for *W*^*S*→*M*^. Choosing a low value of α and low initial weights for *W*^*S*→*M*^ ensures that the activity is driven by the action representation initially and then driven by the lateral weights once the *W*^*S*→*M*^ have been trained sufficiently. The Strio-SOM/Matri-SOM weights (*W*^*S*→*M*^) are thresholded and normalized by their sum to ensure stability.

The matrisomes activity is projected to the direct and indirect pathways by the D1 and D2 neurons of the striatum. In our model, the Matri-SOM activity is modulated by a value difference signal (δ_*V*_). If the agent goes from state *s*^(1)^ to *s*^(2)^, δ_*V*_ is the difference between the value of the two states, i.e., δ_*V*_ = *V*(*s*^(1)^) − *V*(*s*^(2)^).

This value difference signal modulates the switching between the direct and indirect pathways and is thought to be represented by the dopamine signaled by SNc (Chakravarthy and Balasubramani, 2015). The activity of the D1 and D2 neurons are given in Equations (9, 10).

(9) $${Y^{D1}}_{\left[n\right]}=f(\lambda_{D1}\delta_{V}){Y^{M}}_{\left[{n_{s}}^{*}][n\right]}$$

(10) $${Y^{D2}}_{\left[n\right]}=f(\lambda_{D2}\delta_{V}){Y^{M}}_{\left[{n_{s}}^{*}][n\right]}$$

where *f* is a tanh non-linearity and λ_*D*1_ and λ_*D*2_ are the gains of the D1 and D2 neurons respectively. The indirect pathway consisting of the GPe and STN is modeled as network of coupled non-linear oscillators. The dynamics of these oscillators is highly dependent on the input, which constitutes the projections from the D2-expressing neurons of the striatum. The dynamics of GPe is given below:

(11) $$\begin{array}{l} \tau^{GPe}\frac{d{X^{GPe}}_{\left[n\right]}}{dt}=-{X^{GPe}}_{\left[n\right]}-\epsilon^{GPe}{W^{GPe\to GPe}}_{\left[n\right]}{Y^{GPe}}_{\left[n\right]}\\ \;+{W^{STN\to GPe}}_{\left[n\right]}{Y^{STN}}_{\left[n\right]}+{Y^{D2}}_{\left[n\right]} \end{array}$$

(12) $${Y^{GPe}}_{\left[n\right]}=\tanh(\lambda^{GPe}{X^{GPe}}_{\left[n\right]})$$

where *W*^*GPe*→*GPe*^ are the lateral weights within the GPe, ϵ^*GPe*^ is the connection strength, *W*^*STN*→*GPe*^ are the connections between STN and GPe, and λ^*GPe*^ is a non-linear scaling parameter.

The STN layer in the model exhibits correlated activity suppressed for high striatal input, and uncorrelated oscillatory activity for low striatal inputs (Chakravarthy and Balasubramani, 2015). The uncorrelated oscillations of the STN are a key source of exploration for the agent. The dynamics of STN is given below:

(13) $$\begin{array}{l} \tau^{STN}\frac{d{X^{STN}}_{\left[n\right]}}{dt}=-{X^{STN}}_{\left[n\right]}+\epsilon^{STN}{W^{STN\to STN}}_{\left[n\right]}{Y^{STN}}_{\left[n\right]}\\ \;-{W^{GPe\to STN}}_{\left[n\right]}{Y^{GPe}}_{\left[n\right]} \end{array}$$

(14) $${Y^{STN}}_{\left[n\right]}=\tanh(\lambda^{STN}{X^{STN}}_{\left[n\right]})$$

where *W*^*STN*→*STN*^ are the lateral weights within the STN, ϵ^*STN*^ is the connection strength, *W*^*GPe*→*STN*^ are the connections between Gpe and STN and λ^*STN*^ is a non-linear scaling parameter.

The D1 neurons of the striatum and the STN neurons project to the GPi leading to the convergence of the direct and indirect pathways in GPi. In the model, the number of GPi neurons equals number of actions [ = dim(**a**)]. The weights *W*^*D*1 → *GPi*^ and *W*^*STN*→*GPi*^ map the corresponding activities of D1 striatum and STN onto the GPi. The Matri-SOM activity (Y^D1^) corresponding to the chosen action (*a*) (which comes via feedback) is used to train the two sets of weights, *W*^*D*1 → *GPi*^ and *W*^*STN*→*GPi*^ using Hebb's rule. The output of GPi neurons are computed according to Equation (15), and the update for the weights *W*^*D*1 → *GPi*^ and *W*^*STN*→*GPi*^ are done according to Equations (16, 17).

(15) $$\begin{array}{l} {Y^{GPi}}_{\left[n^{\prime }\right]}={W^{D1\to GPi}}_{\left[n^{\prime }][n\right]}{Y^{D1}}_{\left[n\right]}\\ \;-{W^{STN\to GPi}}_{\left[n^{\prime }][n\right]}{Y^{STN}}_{\left[n\right]} \end{array}$$

(16) $$\Delta {W^{D1\to GPi}}_{\left[n][n^{\prime }\right]}=\eta^{D1\to GPi}{Y^{D1}}_{\left[n\right]}{X^{GPi}}_{\left[n^{\prime }\right]}$$

(17) $$\Delta {W^{STN\to GPi}}_{\left[n][n^{\prime }\right]}=\eta^{STN\to GPi}{Y^{STN}}_{\left[n\right]}{X^{GPi}}_{\left[n^{\prime }\right]}$$

The neurons in the GPi project to the thalamus. In our model, action selection takes place in the thalamus, following the integrator-race model (Bogacz, 2007) with thalamic neurons having self-exciting and mutually inhibiting interactions. The thalamic neuron that first crosses a threshold value (*Y*_*thresh*_) determines the action. The thalamic neurons have low initial random activity which converge to a high activity for the chosen action and low values for the others. The dynamics of thalamic neurons is given as:

(18) $${\dot{Y}}_{\left[n\right]}^{Thal}=\sum_{n^{\prime }\in Thal}{W^{Thal}}_{\left[n][n^{\prime }\right]}{Y^{Thal}}_{\left[n^{\prime }\right]}+{Y^{GPi}}_{\left[n\right]}$$

(19) $$a=\{[n]:{Y^{Thal}}_{\left[n\right]}>Y_{thresh}\}$$

This action (*a*) chosen is carried out and the reward (*r*) is obtained. The action chosen is also projected back to the striatum to obtain the activity. Both the action and the reward are used for updates in Equations (6, 8, 16).

### Reinforcement learning in environments with multiple contexts

Standard reinforcement learning techniques are suited for problems where the environment is stationary. However, in some tasks the environment suddenly changes and the agent has to adopt a policy suitable for the new environment. In such a case, the agent identifies the context either using a cue which is representative of the context or using its experience in the preceding trials. One of the techniques to solve problems of the second category is the modular RL framework. In this method, the agent allocates separate modules to separate contexts. Each of the modules has its own copy of the environment in a particular context, represented by an environment feature signal (ρ). This copy is used to generate a responsibility signal, denoted by λ, which indicates how close the current context is to the one represented by the module. Thus, by identifying the module with the highest responsibility signal we can follow the policy developed in that module to solve the problem in an efficient manner.

### Using the striatal modularity to solve modular reinforcement learning tasks

**Figure 3:**
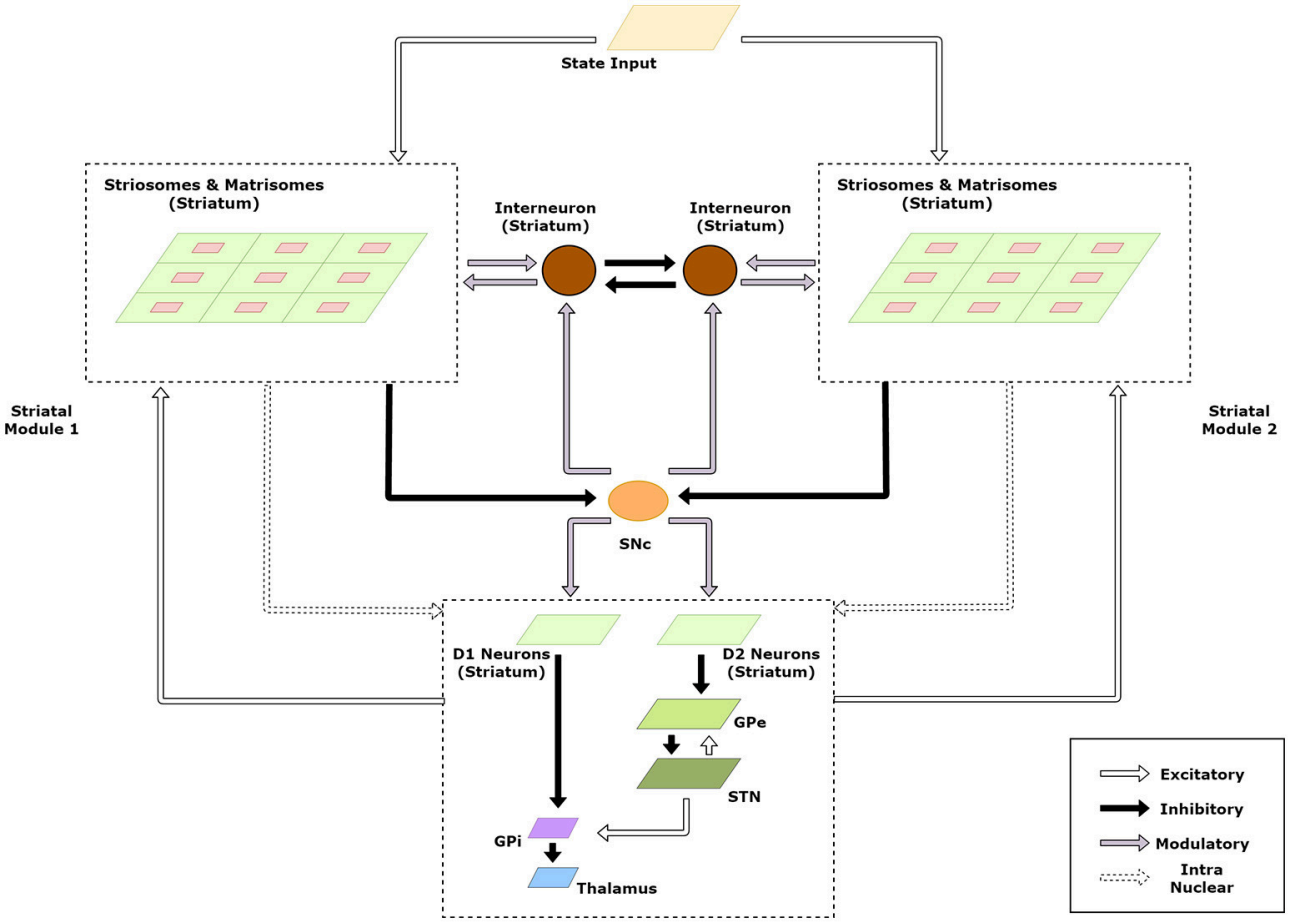
Schematic of the extended model to handle modular RL tasks showing the case with two striatal modules. The state representations of the two modules are used to calculate their respective responsibilities which are then used by the striatal interneurons to choose the appropriate module.

The striatum model developed above forms the basic module capable of solving simple RL tasks. Multiple such modules in the striatum could then be exploited to tackle multi-context tasks using modular RL framework. A schematic of this extended model is given in Figure 3.

We believe that context selection happens at the level of the striatum and the context modulated activity is projected to the downstream nuclei of the BG for further processing. Thus, for clarity, we have expanded the intra-nuclear activity of the striatum in the model schematic (Figure 3). Supposing there are K modules denoted by M_1_, M_2_…, M_K_. We now define the weights and activities in the previous sections for each module and denote {M_i_} with each term associated with module M_i_. Thus, for a module *m*, the following variables undergo a change in notation: *X*^*S*^→*X*^*S*^^, {*m*}^ (Equation 1), *X*^*M*^→*X*^*M*, {*m*}^ (Equation 2), *W*^*S*^→*W*^*S*, {*m*}^ (Equation 3), *W*^*M*^→*W*^*M*^^, {*m*}^ (Equation 4), *V*(*s*) → *V*^{*m*}^(*s*) (Equation 5), *W*^*Str*→*SNc*^→*W*^*Str*→*SNc*, {*m*}^ (Equation 6), *X*^*M*^→*X*^*M*, {*m*}^ (Equation 7), *W*^*S*→*M*^→*W*^*S*→*M*, {*m*}^ (Equation 8).

We propose that in addition to the value of the state *s*, the activity of the Strio-SOM also projects to the SNc to represent the environment feature signal (ρ^{*m*}^). The weights of these projections are denoted as $W_{\rho }^{Str\to SNc}$^, {*m*}^ and are trained using the signal from SNc which is representative of context prediction error (δ^*^). The corresponding equations are given in Equations (20, 21). The context prediction error is calculated as δ^*^ = *r* − ρ^{*m*}^(*s*)

(20) $$\rho^{\left\{m\right\}}(s)=\sum_{\forall n}{{W_{\rho }}^{Str\to SNc,\{m\}}}_{\left[n\right]}{X^{S,\{m\}}}_{\left[n\right]}$$

(21) $$\Delta {W_{\rho }}^{Str\to SNc,{\left\{m\right\}}_{\left[n\right]}}={\eta_{\rho }}^{Str\to SNc}\delta^{*}{X^{S,\{m\}}}_{\left[n\right]}$$

We believe that the selection of the appropriate module for the context is guided by the striatal interneurons. In our model, the activity of the interneurons represents the responsibility signal for each module, denoted by λ^{*m*}^ for module m. In a given state *s*, the inter-neurons compete among themselves and the one with the highest λ chooses the module responsible for deciding the action in that state. Let the winning module in the state *s* be denoted by m^*^. This module guides the projection to the direct and indirect pathway (Equations 9, 10) as given in Equations (22, 23).

(22) $${Y^{D1}}_{\left[n\right]}=f(\lambda_{D1}\delta_{V}){Y^{M,\{m^{*}\}}}_{\left[{n_{s}}^{*}][n\right]}$$

(23) $${Y^{D2}}_{\left[n\right]}=f(\lambda_{D2}\delta_{V}){Y^{M,\{m^{*}\}}}_{\left[{n_{s}}^{*}][n\right]}$$

Following this stage, the equations governing the signal flow are same as that in the previous section. The weight updates in the striatum are however done only to the module m^*^.

The dynamics of the responsibility signal is given in Equation (24)

(24) $$\dot{\lambda }=-\lambda -\alpha_{\lambda }{\left(\delta^{*}\right)}^{2}$$

where α_λ_ controls the influence of context prediction error on the responsibility signal and δ^*^ is the context prediction error.

## Results

### Modeling the microanatomy of the striatum

We use a grid-world problem as a preliminary benchmark to test our model. The grid is of size 10 × 10 and the agent can take one of the four actions—up, down, right and left in a state. A reward is placed at one of the corners of the maze. The goal of the task is to make the model (agent) learn to reach this goal. We use the terms model and agent interchangeably in these sections since we use the model as a reinforcement learning agent in the various tasks. We used a 10 × 10 Strio-SOM to represent the state space and a 3 × 3 Matri-SOM, associated with each of the Strio-SOM neurons, for representing the action space.

**Figure 4:**
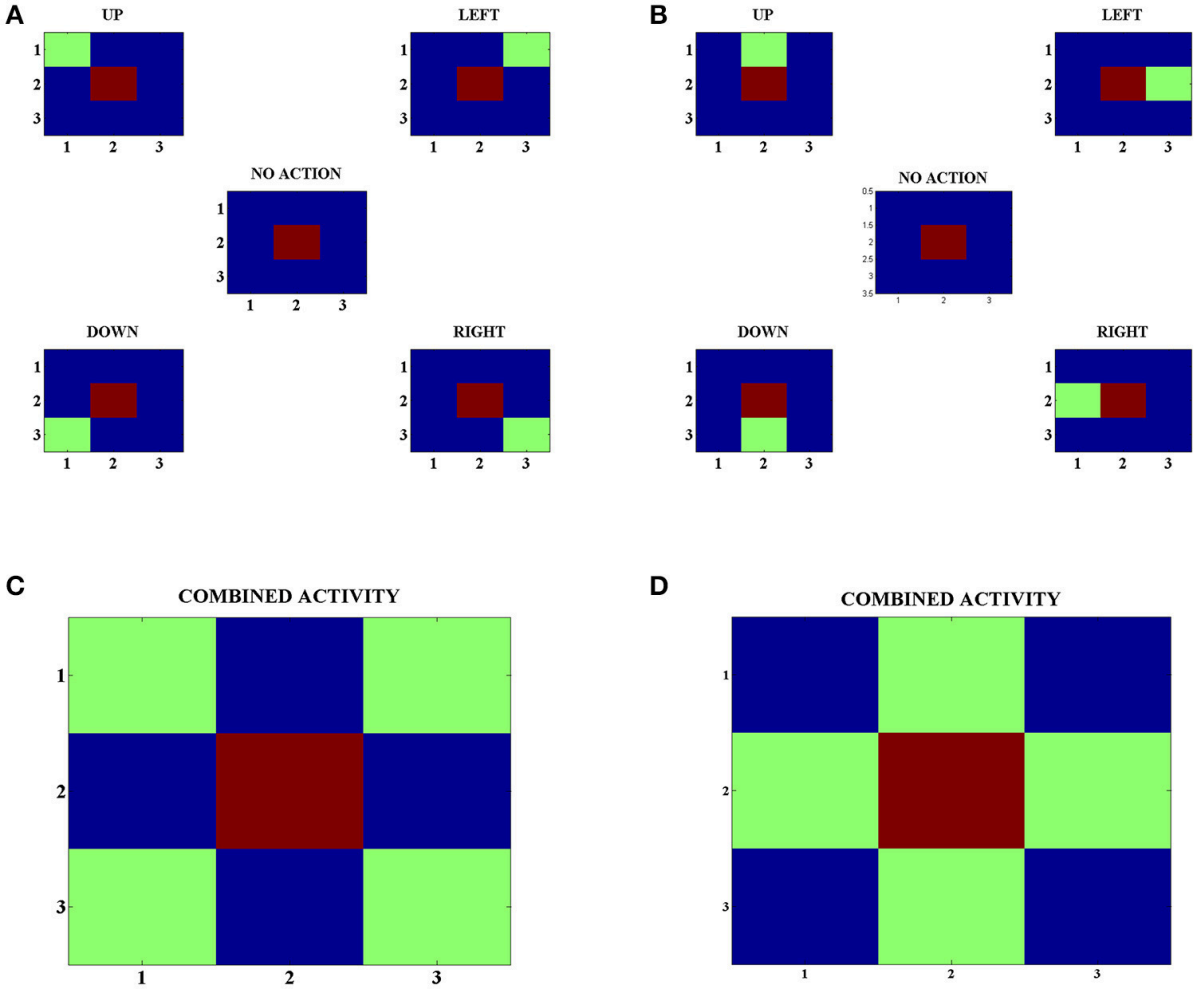
(A) Activity of the Strio-SOM and the corresponding Matri-SOM neurons for different actions in a state. The center map shows only the activity of the Strio-SOM in the absence of any action and the other four maps in the corners show the activity of the Strio-SOM and the four possible Matri-SOM neurons that best respond to the particular action. **(B)** Same as **(A)** for another state. **(C)** Combined activity for all the action pairs in **(A)**. Shows one configuration of the center-surround mapping. **(D)** Combined activity for all the action pairs in (B). Shows another configuration of the center-surround mapping.

In order to develop these representations, we make the agents explore various states and choose random actions in those states. Following this, we look at the neuron with the highest activity in the Strio-SOM for a particular state and the neurons with the highest activity for each action in the corresponding Matri-SOM for that state (Figures 4A,B). Upon looking at the combined Matri-SOM activity for all the actions, we observed predominantly two different configurations of the center-surround mapping (Figures 4C,D).

### Reinforcement learning in a single context gridworld task

**Figure 5:**
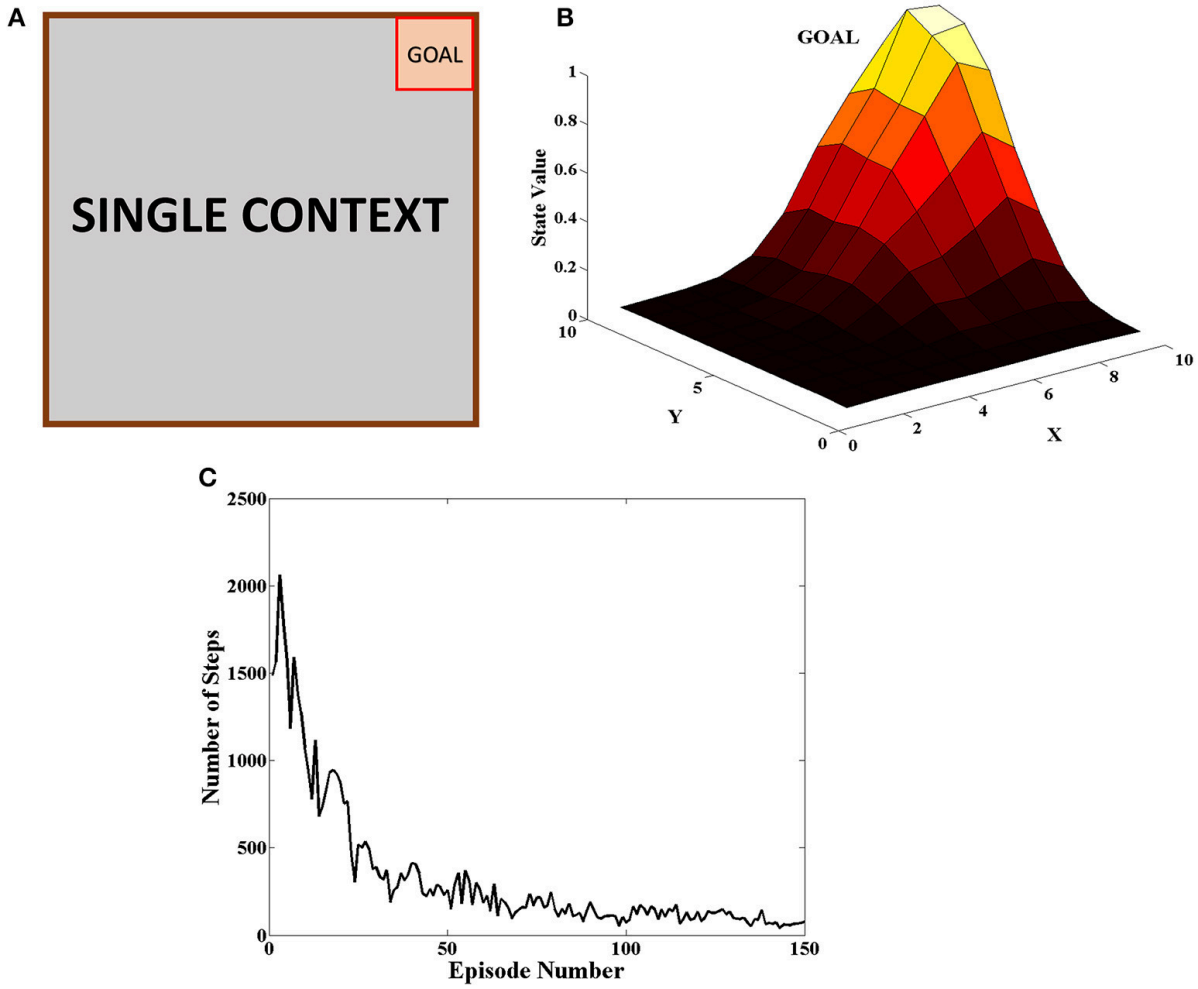
**(A)** Schematic of the grid-world used in the task. A goal is located at the top right corner of the grid **(B)** State value map estimated by the agent at different spatial locations. We can see that the state value peaks at the goal location. **(C)** Plot of the Number of Steps taken by the agent in each episode averaged across 50 independent sessions. We see that the number of steps reduces as the agent learns across episodes.

The goal was placed at the top right of the grid as seen in Figure 5A. The agent received a reward of +20 when it reached the goal and 0 for all the other steps. At the beginning of an episode, the agent started at random and the episode ended when the agent reached the goal or when it reached the upper limit on number of steps allowed in the episode. The agent carried on the task for 150 episodes. This procedure was carried out for 50 independent sessions and the mean number of steps to reach the goal in a particular episode was plotted in Figure 5C. The heat map of the state value function (Equation 5) estimated by the agent at different spatial locations is given in Figure 5B and peaks at the goal location. This combined with the fact that number of steps reduces as the episodes progress indicate that the agent is able to learn the single context task. The various parameter values for this task are given in Table 1.

**Table 1:** Parameter values for single context and multi context tasks.

**Parameter**	**Value**
**Strio-SOM Dimension (*m*_1_ × *n*_1_)**	**10 × 10**
σ_*S*_	0.01
η_*S*_	0.4
γ	0.97
α	0.1
λ_*D*1_	1
τ^*GPe*^	3
ϵ^*GPe*^	−0.01
λ^*GPe*^	3
η^*D*1 → *GPi*^	0.01
*Y*_*thresh*_	1
α_λ_	0.8
**Matri-SOM Dimension (*m_2_ × n_2_*)**	**3 × 3**
σ_*M*_	0.1
η_*M*_	0.4
η^*Str*→*SNc*^	0.1
η^*S*→*M*^	0.1
λ_*D*2_	−1
τ^*STN*^	1
ϵ^*STN*^	0.01
λ^*STN*^	3
η^*STN*→*GPi*^	0.01
$\eta_{\rho }^{Str\to SNc}$	0.1

### Reinforcement learning in a multi-context grid-world problem

**Figure 6:**
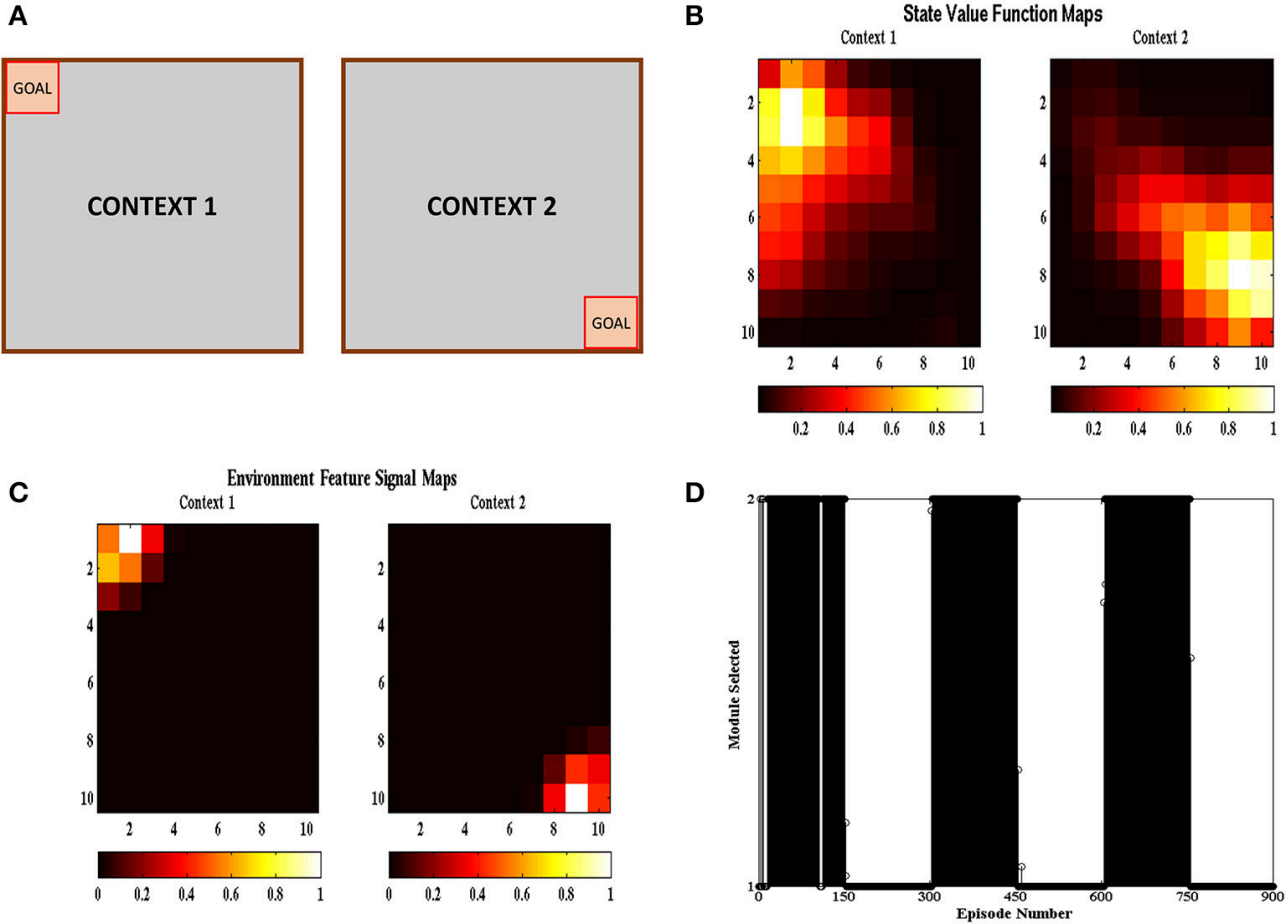
**(A)** Schematic of the gridworld used in the task. A goal is switched between the top left and bottom right corner every 150 episodes. **(B)** State value map estimated by the agent at different spatial locations across different contexts. We can see that the state value peaks at the goal location corresponding to the context. **(C)** Environment Feature Signal maps estimated by the agent at different spatial locations across different contexts. We can see that the state value peaks at the goal location corresponding to the context. **(D)** Modules chosen by the agent at different episodes. We can see that the module chosen switched with change in context indicating that the agent is able to identify the context it is currently present in.

In the multi context grid-world tasks, the agent had to reach the goal like the previous section but the goal location changed after a certain number of episodes. The goal was present either at the top right corner or at the bottom left corner as shown in Figure 6A. The goal was switched to the other location after 150 episodes. The task was carried out in 50 independent sessions with each session containing 900 episodes. The parameters used have the same values as given in Table 1. Figure 6B shows the value function (Equation 5) heat map and Figure 6C shows the environment feature signal (Equation 20) heat map estimated by the agent for the two contexts. We can observe that the agent is able to learn these values for both the contexts. Figure 6D shows the context chosen by the agent in different episodes and we can observe that the agent is able to switch context in sync with the switch in reward distribution. These results illustrate that the agent is able to successfully identify the context it is presently in, and complete the corresponding grid-world task.

**Figure 7:**
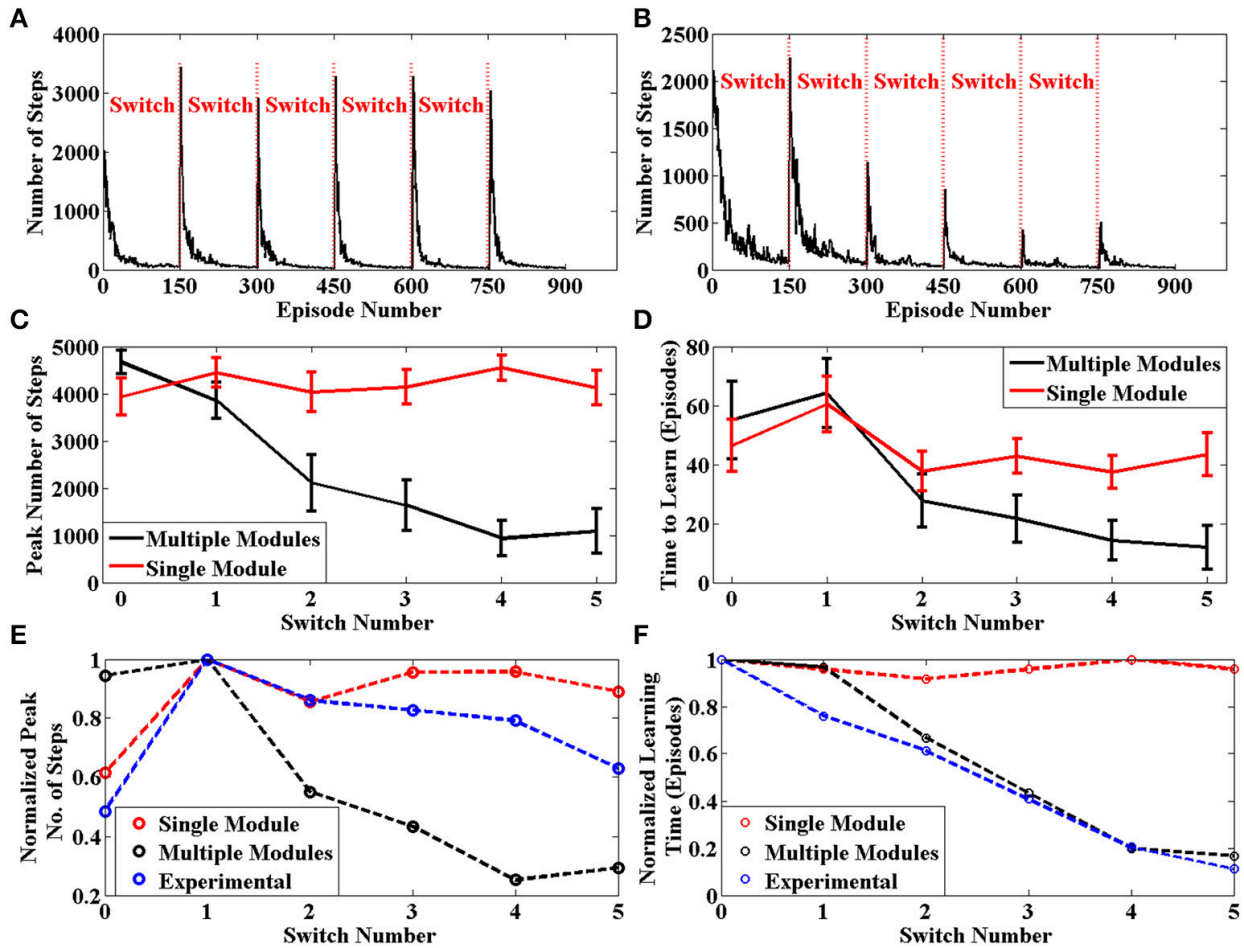
(A) Plot of Number of Steps taken by the single module agent in each episode averaged across 50 independent sessions. We see that the agent needs to relearn after each context switch **(B)** Plot of Number of Steps taken by the multi module agent in each episode averaged across 50 independent sessions. We see that the agent efficiently switches modules after each context switch **(C)** Peak number of steps needed to reach the goal after a context switch averaged across 50 sessions. **(D)** Number of episodes for the number of steps required to reach the goal to go below a certain threshold averaged across 50 sessions **(E)** Peak value for the average number of steps needed to reach the goal after a context switch. The experimental values have been adapted from Brunswik (1939) **(D)** Number of episodes for the average number of steps required to reach the goal to go below a certain threshold. The experimental values have been adapted from Brunswik (1939).

The average number of steps required by the agent to reach the goal for each episode across 50 sessions is given in Figure 7B. The same plot for an agent with only a single module is given in Figure 7A. We can clearly see that the learning is more efficient for multi module agent as compared to the single module case. In order to quantify this improvement, we use two values to measure the agent's performance after a context switch. These are the peak number of steps to reach the goal after a context switch and the number of episodes for the number of steps needed to go below a certain threshold. We calculate these two values for each context switch in a session. These values are averaged across sessions and presented in Figures 7C,D, respectively. In both cases, we see that the multi module agent is better than the single module agent for solving the task. We use these measures to compare the model against experimental data in Brunswik (1939). Since we only have the average performance across sessions available in the reference, we calculate the corresponding values from our model and present these for the single module, multi module and the experimental case in Figures 7E,F, respectively. We can observe that multi module results have a similar trend to the experimental results as compared to the single module model, thus demonstrating that the BG could be using the modular architecture of the striatum to solve context switching tasks.

## Discussion

We have proposed a network model of BG incorporating a computational framework to capture the microanatomy of the striatum. Our model shares features with existing models of BG designed to solve reinforcement learning (RL) tasks. In addition to solving RL tasks, our model exploits the modularity of the striatum to solve tasks with varying reward distributions in multiple contexts.

### Striosome-matrisome dynamics with their dopaminergic projections

Our model is based on the assumption that striosomes map state information and matrisomes map action information. Earlier results suggest that the striosomes receive input from the orbitofrontal cortex (Eblen and Graybiel, 1995) known for coding reward related states (Wilson et al., 2014). Matrisomes receive connections from primary motor and somatosensory cortices and could have action representations (Flaherty and Graybiel, 1994), thereby supporting the assumptions of our model. Anatomical studies show that striosome medium spiny neurons (MSNs) project directly to SNc (Fujiyama et al., 2011; Lanciego et al., 2012; Smith et al., 2016). We believe that these projections could code for the state value of the agent as seen from the Strio-SOM to SNc connections in our model.

We propose that the striosome neurons influence the behavior of the surrounding matrisome neurons. Earlier results show that fast spiking interneurons (FSIs) and persistent and low-threshold spike (PLTS) interneurons are anatomically suitable candidates for this role since they branch across the patch and matrix (Gittis and Kreitzer, 2012). We believe that the dopaminergic projections to these interneurons (Bracci et al., 2002) could allow the striosome to bias the surrounding matrisome activity toward a preferred action. This is done by the thalamic feedback which drives the matrisome activity to the action chosen which is then reinforced by the prediction error signaled by the SNc. To our knowledge, this modulation (Equation 8) is a unique feature to our model and gives a biologically plausible mechanism to perform Q-learning. This is also supported by experiments which indicate that the striatum contributes to action selection by biasing its output toward the most desirable action (Samejima et al., 2005; Hikosaka et al., 2006).

### Mapping representations to action primitives

Striatal MSN recordings show that they encode action representations and are modulated by the expected reward for the actions (Isomura et al., 2013). Our model agrees with this as both the Matri-SOM D1 and D2 neurons represent the action space and are correspondingly modulated by the TD error which is representative of the expected reward. Experiments also show activity in the MSNs corresponding to the outcome of the chosen action (Kim et al., 2009). We believe again that this could be the signal required to bias the activity of the striatal MSN as seen in the model (Equation 8).

GPi forms the output nucleus of the BG and receives projections from Striatal MSNs through the direct and indirect pathways. Lesion studies show that GPi controls movement by inhibitory projections to the thalamus and lesioning GPi impairs motor responses (Baunez and Gubellini, 2010). Experiments also show that in the executive part of the task, the GPi activity is strongly related to the action performed (Pasquereau et al., 2007).

We propose that the connections from striatal D1 MSNs and STN to the GPi map the projections from action representations to action primitives. We believe that this mapping provides a flexible method to switch different action primitives for the same representations and vice versa, providing a plausible mechanism of adaptation in learning. Experiments show evidence of transformation of action information seen as higher degree of correlation in GPi activity as it passes from striatum to the GPi (Garenne et al., 2011).

### Contextual learning and striatal modularity

Contextual Learning refers to the ability of the agent to adapt and learn in different contexts. Some earlier operant conditioning experiments in such tasks have an explicit indication of contexts (different room or color for each context) using which the agent can choose its actions (Bouton and King, 1983; Bouton and Peck, 1989). In such tasks, the agent shows renewal upon context switching indicating a mechanism for context identification. Experimental results indicate that the BG encodes the context as well as the choices in those contexts (Garenne et al., 2011).

A recent study (Amemori et al., 2011) hypothesized that the modular architecture of the striatum makes it a suitable candidate for solving multi-context RL problems. We build on this by providing a computational neural model for the same. We describe the plausible correlates for computing the necessary variables for solving multi-context problems using a modular setting. The context prediction signal is very similar to a state value and we propose that neurons in the SNc code for this signal as well (Tobler et al., 2005). In our model this is represented by the projections from Strio-SOM to the SNc. There is also a need for a reward prediction variance signal or a risk signal. Dopamine in the midbrain is proposed to also represent the risk component in the environment (Schultz, 2010). In addition, it has been proposed that serotonin activity in the striatum correlates to risk or reward variance, just as dopamine codes for reward prediction error (Balasubramani et al., 2015).

We propose that the module selection and switching in different contexts could be carried out by TANs. TANs exert a strong influence on striatal information processing and lesioning inputs to TANs impair learning after a change in reward distribution (Ragozzino et al., 2002). In our model, the TANs compete with each other and select the module appropriate for the task. Experiments support this hypothesis by showing that TANs can compete with each other using inhibitory connections similar to the model (Sullivan et al., 2008) and can cause widespread inhibition of MSNs by activating a GABAergic subpopulation (English et al., 2012). Another plausible method for context switching by TANs is by producing acetylcholine (ACh) which can inhibit targeted MSNs. Dynamic changes in ACh output in the medial striatum (Ragozzino and Choi, 2004) during reversal learning supports this claim.

### Behavioral observations

Several behavioral processes were also observed from the results of the experiments on the model. We saw in Figure 6B that the agent increases its down and right actions when the goal is placed at the bottom right corner. The agent thus exhibits acquisition (Graham and Gagné, 1940) since it strengthens certain actions over the others based on the reinforcement given. We saw in Figure 6D, that once the context has changed, the agent stops choosing the initial preferred response. This demonstrated extinction (Graham and Gagné, 1940) since the behavior associated with a certain task gets elimininated when the reinforcement associated is removed. The experiments also indicate that the agent is able to show stimulus generalization and stimulus discrimination as the agent is able to distinguish between two different contexts which are two distinct stimuli (Till and Priluck, 2000). Also the value function peaks where the goal is given, therefore goals which are near each other will have similar value profiles. From Figure 7B, we saw that after two changes when the initial context reappears, the agent is able to bring back the policy learnt almost immediately exhibiting spontaneous recovery (Graham and Gagné, 1940) referring to the reappearance and faster relearning of a previously extinguished response.

## Limitations and future work

In a variable environment, there are two types of uncertainty—expected uncertainty/Risk which refers to the uncertainty even after full learning and unexpected uncertainty which is related to a sudden change in the environment. While the latter is tested in our model with the help of context switches, the rewards are certain and this makes the learning and module switching easier. However, the next step would be to look at harder problems where the rewards are also stochastic. In this case, the ability to detect change in context no longer remains trivial and would be an interesting problem to study.

The experimental validation in such tasks becomes very challenging due to the high number of states and trials required. While the grid world is a natural problem for testing RL frameworks, the number of trials continuously for a real animal is limited. Our model requires around 900 trials despite the various simplifying assumptions which is very taxing for the animal. Thus, there is a need to look at simpler tasks where we can test the model and the animal on various intricacies of the problem.

## Author contributions

SS, VM: Conceived, developed the model and prepared the manuscript. VC: Conceived the model and prepared the manuscript.

### Conflict of interest statement

The authors declare that the research was conducted in the absence of any commercial or financial relationships that could be construed as a potential conflict of interest.
